# Electrodialysis Tartrate Stabilization of Wine Materials: Fouling and a New Approach to the Cleaning of Aliphatic Anion-Exchange Membranes

**DOI:** 10.3390/membranes12121187

**Published:** 2022-11-25

**Authors:** Kseniia Tsygurina, Evgeniia Pasechnaya, Daria Chuprynina, Karina Melkonyan, Tatyana Rusinova, Victor Nikonenko, Natalia Pismenskaya

**Affiliations:** 1 Membrane Institute, Kuban State University, 350040 Krasnodar, Russia; 2 Department of Analytical Chemistry, Kuban State University, 350040 Krasnodar, Russia; 3 Central Research Laboratory, Kuban State Medical University, 350040 Krasnodar, Russia

**Keywords:** aliphatic ion exchange membrane, electrodialysis, tartrate stabilization, fouling, cleaning

## Abstract

Electrodialysis (ED) is an attractive method of tartrate stabilization of wine due to its rapidity and reagentlessness. At the same time, fouling of ion-exchange membranes by the components of wine materials is still an unsolved problem. The effect of ethanol, polyphenols (mainly anthocyanins and proanthocyanidins) and saccharides (fructose) on the fouling of aliphatic ion-exchange membranes CJMA-6 and CJMC-5 (manufactured by Hefei Chemjoy Polymer Materials Co. Ltd., Hefei, China) was analyzed using model solutions. It was shown that the mechanism and consequences of fouling are different in the absence of an electric field and during electrodialysis. In particular, a layer of colloidal particles is deposited on the surface of the CJMA-6 anion-exchange membrane in underlimiting current modes. Its thickness increases with increasing current density, apparently due to the implementation of a trap mechanism involving tartaric acid anions, as well as protons, which are products of water splitting and “acid dissociation”. A successful attempt was made to clean CJMA-6 in operando by pumping a water-alcohol solution of KCl through the desalination compartment and changing electric field direction. It has been established that such a cleaning process suppresses the subsequent biofouling of ion-exchange membranes. In addition, selective recovery of polyphenols with high antioxidant activity is possible.

## 1. Introduction

In the food industry, there has been a trend of transition to the circular economy in recent years. In the case of the winery industry, as well as the processing of vegetables and fruits, the circular economy suggests implementing the recovery of bioactive ingredients such as polyphenols with antioxidant activity, vitamins, enzymes, oils and carotenoids, as well as polybasic organic acids from the generated waste. These substances can be used in natural food supplements and innovative functional products [1]. In particular, electrodialysis (ED) has been used for the valorization of distilled vinasses from the winery industry by means of tartaric acid recovery [2]. Application of a conventional membrane stack with the CEM-AEM-CEM configuration (AEM, CEM are anion and cation-exchange membrane correspondently) allows concentrating potassium hydrogen tartrate (KHT) two times compared to distilled vinasses, and then producing 9.9 g/L of tartaric acid (H_2_T) and 5.6 g/L of potassium hydroxide (KOH) using membrane stack containing bipolar membranes. These new approaches are based on already known processes, in particular tartrate stabilization of wine using ED, which has been developed since the 1980s–1990s of the 20th century [3,4]. It is now in common use [5] and has proven to be competitive [6,7] with traditional methods such as low-temperature stabilization [8,9] or the addition of carboxymethyl cellulose, mannoproteins and metatartaric acid. Potassium polyaspartate and other chemical reagents [6,10]. It has been shown [11] that overall electrical energy consumption for ED stabilization (0.21 kWh/hL) is eight times lower compared with cold stabilization. In addition, ED reagentless pH control due to water splitting at the AEM/diluted solution or AEM/CEM interface is becoming increasingly popular in the wine and juice industry [12,13]. The advantages of ED stabilization and reagentless pH regulation are rapidity; the possibility of automation and implementation of the process in a continuous mode; small production areas occupied by ED set-ups and their mobility; and the use of a limited amount of chemicals, which are mainly consumed to counteract the fouling of ion-exchange membranes by wine components [14,15]. Acids [16,17], alkalis [18], oxidants (hypochlorides, peracetic acids, P3 Active Oxonia^®^ solutions) [19] or mixtures of polar and non-polar organic solvents are usually such cleaning chemicals [20]. This cleaning provides an increase in the life cycle of certain types of membranes up to several years. However, then the membrane stacks have to be partially or completely changed due to the destruction of ion-exchange materials, inert filler or reinforcing materials. Therefore, cleaning and replacement of membranes has led to an increase in the cost of ED processes by 40% or more in the food industry [15].

An alternative to chemical cleaning is using membranes with anti-fouling properties [21,22] or special current modes. For example, the pulsed electric field (PEF) mode is used for reducing the fouling and scaling process. An electrical pulse of direct continuous current (or potential drop) alternates with a pause during which the current is zero in the PEF mode [23]. This mode is applied for ED processing of proteins [24], humic acids [25], cranberry [26] and other juices [27], extraction or separation of blood serum components [28,29], etc. [30,31]. It seems that the PEF mode produces good results. In the case of sweet whey, the PEF mode promotes shifting the pH value to a lower region and leads to reducing electrostatic interactions of proteins with membrane material [32]. However, the mechanisms of the cleaning process for other foulants are not clear yet, and the PEF parameters are selected empirically for each specific foulant.

Hansima et al. discuss in a review [33] reverse electrodialysis (EDR) for removing hardness, salinity, charged organics and even turbidity from water, even with high saturation and Langelier indexes. EDR is characterized by a periodic change in the polarity of the electric field [34]. As a rule, flow rate and component composition of solutions pumping through compartments of an electrodialyzer remain unchanged [35,36]. Most often, EDR is effective in counteraction of scaling (precipitation of minerals on membrane surface) [37]. Recently, there is increasing evidence that EDR is a promising method for counteracting the fouling of humic acids, antibiotics and other substances that have a similar chemical structure to anthocyanins and proanthocyanidins (components of wine) [36,38]. At the same time counteraction of fouling in the ED processing of wine requires a combined approach, including both the use of special current modes and chemical reagents.

Reviews [14,15] summarize the achievements of recent years in the study of fouling mechanisms. For example, it has been found that one of the main causes of fouling is electrostatic interactions between fixed groups of ion-exchange membranes and components of wine materials or juices [39,40,41]. Moreover, a number of ampholyte substances (anthocyanins, amino acids, anions of polybasic acids, etc.) can change their electric charge when entering the membrane compared to the one they have in the processed liquid. These changes are caused by the acidification of the internal CEM solution and the alkalinization of the internal AEM solution compared to the external solution due to the Donnan exclusion of coions, products of ampholyte protonation-deprotonation reactions [39]. Another reason is π-π (stacking) interactions of aromatic rings of anthocyanins (Ant), proanthocyanidins (PACs) and other polyphenols components of wine, juice or tea with the aromatic matrix of membranes [42,43,44,45]. In addition, polyphenols form hydrogen bonds, as well as complexes and colloidal particles, with many components of wine [46,47]. Therefore, even a small amount of these substances can become a trigger for membrane fouling.

It should be noted that, until recently, aromatic membranes, in particular, AMX-Sb, CMX-Sb (manufactured by Astom, Yamaguchi Japan), were mainly used in the ED processing of wine materials and juices [5,10,42,48]. In recent years, membranes with an aliphatic matrix have appeared (manufacturers Fujifilm, Tilburg, The Netherlands; ChemJoi Ltd., Hefei, China, etc.), which are increasingly used in ED, including for the extraction of tartrates and deacidification of juices [2,16,43]. The study of the fouling of such membranes during the ED processing of juices and wines is at an early stage [43,49,50]. In addition, when studying the consequences of fouling, attention is mainly paid to the ion-exchange capacity, conductivity as well as the diffusion permeability of membranes [42,51,52] and, to a lesser extent, the behavior of ion-exchange membranes in an electric field [53]. This is probably why there is still no comprehensive understanding of the role of each of the main components of wine (tartaric acid anions, ethyl alcohol, polyphenols, sugars) in fouling, as well as the effect of fouling on ED performance in winemaking or juice production.

Our study focuses on the study of the fouling of aliphatic membranes CJMC 5 and CJMA-6 and the effect of this phenomenon on the mass transfer characteristics and energy consumption in the process of tartrate ED stabilization of model solutions of wine materials. We evaluated which of the components of wine (tartrates, ethyl alcohol, anthocyanins and proanthocyanidins or fructose) has a greater effect on the current–voltage characteristics of the anion-exchange membrane and the degree of removal of tartrates. In addition, the in operando method of cleaning this membrane with an acidified water–ethanol salt solution and using the electric field will be tested.

## 2. Materials and Methods

### 2.1. Membranes

Quasi-homogeneous ion-exchange membranes CJMA-6 and CJMC-5 manufactured by Hefei Chemjoy Polymer Materials Co. Ltd., Hefei, China, formed the diluate and concentrate compartments of the membrane stack of a flow laboratory electrodialysis cell. These membranes are produced by the casting method. First, the monomers are polymerized and an aliphatic matrix is obtained. Then the high-molecular polymer is cross-linked with cross-agents and fixed (functional) groups are introduced [54]. Manufacturers reinforced these membranes with polyethylene terephthalate cloth to provide mechanical strength.

Heterogeneous anion-exchange membranes MA-41 (manufacturer Shchekinoazot, Shchekino, Russia) form cathode and anode electrode compartments with platinum polarizing electrodes (Pt). MA-41 is produced by hot rolling powder of anion-exchange resin AB-17-8 and low-pressure polyethylene, followed by reinforcement with a polyamide net. 

Some characteristics of the membranes used are presented in Table 1.

### 2.2. Solutions

Table 2 contains designations, composition and characteristics of model solutions of wine materials. The detailed composition of the model solutions and red wine (for comparison) is presented in Supplementary Materials (SM), Table S1. These solutions were pumped through the DC desalination compartment in experiments on ED recovery of tartrates (solutions 1–4) and in a blank experiment (solution 5) to detail the mechanism of ion-exchange membrane fouling.

The pH, electrical conductivity and concentrations of the components of these solutions are typical for red wine [47,56]. Of the more than 600 components that wines contain, those that are the target of tartrate stabilization, as well as those that can significantly affect the fouling of ion-exchange membranes, have been selected [39,42,50]. The mole fractions of tartaric acid species in model solutions were calculated using pH values and equilibrium dissociation constants of tartaric acid for the 1st and 2nd steps [57]. The structure of anthocyanins (Ant) and color of their solutions depending on the pH is shown in SM, Figures S1 and S2. Figures S3, S4 and S5 show the structure of proanthocyanidins (PACs), fructose and tartaric acid (H_2_T), respectively. The distribution of tartaric acid species (in mole fractions) depending on the pH of the solution is shown in Figure S6. In membrane cleaning experiments, the DC circuit was filled with a cleaning solution (pH 3.25) containing 0.005 M KCl and 10% ethanol. A 0.005 M KCl solution with pH 3.25 was pumped through all other compartments of the ED cell in all experiments.

To prepare the solutions, we used crystalline salts of tartaric acid of analytical grade (manufacturer JSC Vekton, Saint Petersburg, Russia) and potassium chloride of chemical purity grade (manufacturer Mikhailovsky Plant of Chemical Reagents, Barnaul, Russia) as well as fructose (manufacturer Tate & Lyle, Boleraz, Slovakia s.r.o). An extract from grape pulp (producer Etol, Škofja Vas, Slovenia) was a source of Ant and proanthocyanidins (PACs). All solutions were prepared using distilled water with electrical conductivity 3.61 ± 0.01 µS/cm and pH 5.04 ± 0.01 at 25 °C.

### 2.3. Electrodialysis Unit and Experimental Technique

**Figure 1:**
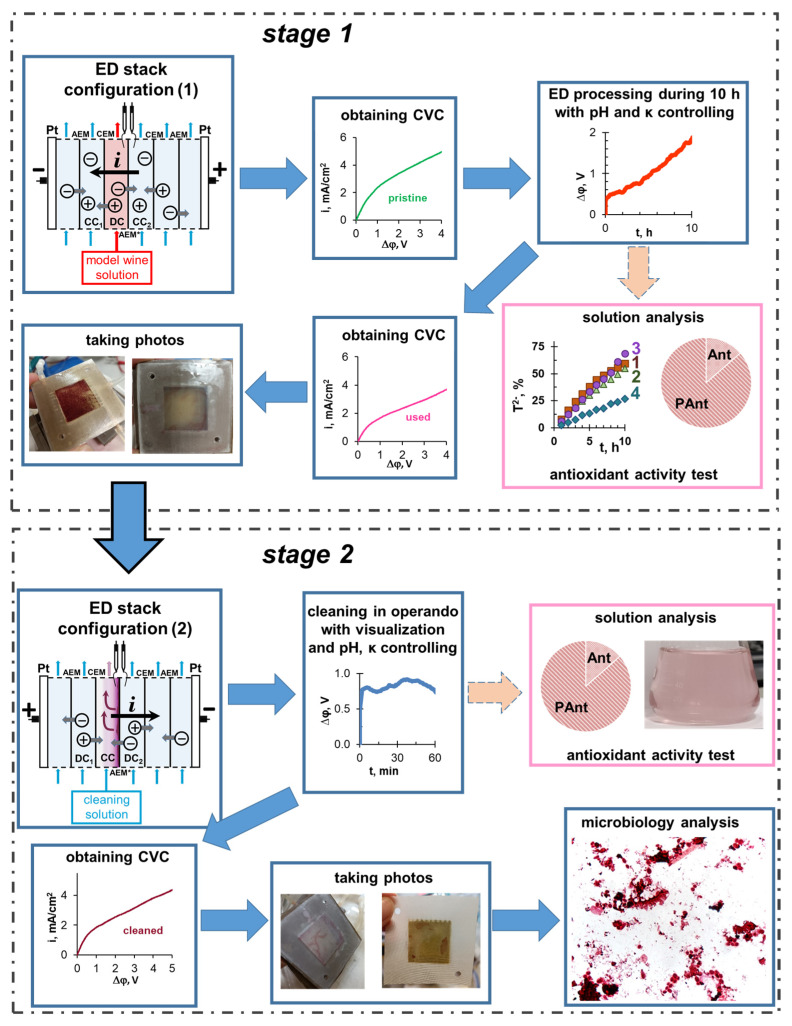
Scheme of the protocol for the implementation of experiments.

**Figure 2:**
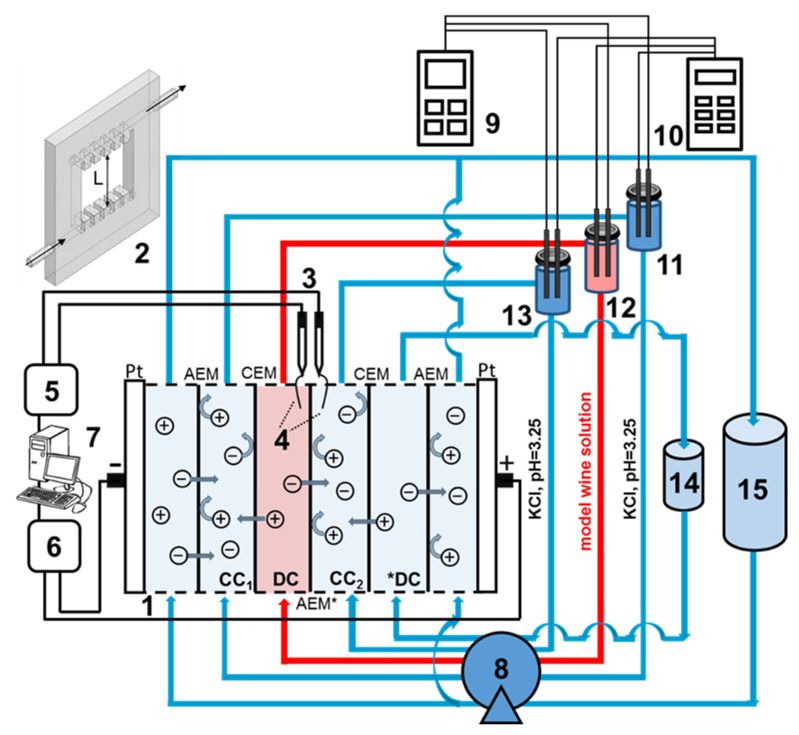
Scheme of the experimental setup: 1—six-compartment flow ED cell, where AEM is an anion-exchange membrane, CEM is a cation-exchange membrane, AEM* is an anion-exchange membrane under study, CC_1_ and CC_2_ are concentration compartments 1 and 2, respectively, DC is the studied desalination compartment; *DC is an auxiliary desalination compartment; 2 is a schematic representation of the plastic frame of the cell and the solution flows through it, where L is desalination length; 3—Ag/AgCl electrodes; 4—Luggin’s capillaries; 5—multimeter; 6—current source; 7—personal computer; 8—peristaltic pump; 9—pH meter; 10—conductometer; 11–13—intermediate tanks in the DC, CC_1_ and CC_2_ circuits; 14—intermediate tank for the solution circulating in the *DC circuit; 15—intermediate tank for the solution circulating in the EC circuits.

The experimental protocol is schematically shown in Figure 1. Figure 2 represents the scheme of the experimental setup. The six-compartment laboratory electrodialysis cell 1 has a working area of polarizing platinum electrodes (Pt) and each of the ion-exchange membranes is equal to 7.29 ± 0.01 cm^2^. The length of the solution desalination path is 2.70 ± 0.01 cm. Plastic frames 2, equipped with comb devices for input and output of the solution to ensure the laminar hydrodynamic regime of the pumped liquid, separate all membranes. The electrode compartments (EC) formed by frames and membranes, as well as the desalination (DC) and concentration (CC) compartments, do not contain spacers. The intermembrane distance is 0.66 ± 0.01 cm. CC_1_, CC_2_ and DC are fed from tanks 11, 12 and 13. The circuits of these compartments contain 150 ± 1 cm^3^ of solution. Auxiliary DC was fed from tank 14 with a volume of 1000 ± 1 cm^3^. Both SEs were fed from the same container 15 with a volume of 5000 ± 1 cm^3^. The average linear pumping velocity of solutions was 0.42 cm/s (DC, CC_1_ and CC_2_) and 1.68 cm/s (EC). 

In experiments on ED recovery of tartrates (stage 1), sensors connected to the Expert-001 and Expert-002 devices and immersed in containers 11, 12, 13 measured the pH and electrical conductivity values in the DC, CC_1_ and CC_2_ circuits every 20 min during ED processing of the model solution. In addition, every 60 min, 1.25 cm^3^ of the solution was taken from tanks 11, 12 and 13. The concentration of the studied ions in the solutions was determined using a DIONEX ICS-3000 (Sunnyvale, CA, USA) chromatographic system with a conductometric detector and a background signal suppression unit. The concentrations were calculated using calibration curves. The concentrations of Ant and PACs were determined using an ECOVIEW UV-1800 spectrophotometer (Shanghai Mapada Instruments Co., LTD, Shanghai, China) according to the procedures described in [58,59], respectively.

The value of the current density applied to the cell during ED was 1.22 ± 0.01 mA/cm^2^ and did not exceed the value of the limiting current found by crossing the tangents to the initial section and the section of the inclined plateau of the current-voltage curve (CVC) (see Section 3.3). CVC was obtained before and immediately after the implementation of electrodialysis. Each ED experiment lasted 10 h. In addition, in a special experiment, ED processing of model solution 3 was carried out in the overlimiting current mode at 3.26 mA/cm^2^ for 18 h.

After the implementation of ED and obtaining the CVC, the ED cell was disassembled. The surfaces of the membranes facing the desalination compartment were photographed or studied using a SOPTOP research class laboratory optical microscope (model CX40M, NINGBO SUNNY INSTRUMENT CO. LTD, Ningbo, China).

Cleaning in operando experiments (step 2) were performed with the membranes used in the ED processing of model solution 4 (Table 2). The ED cell was preliminarily cleaned from the components of the model solution. The DC circuit was filled with a cleaning solution (see Section 2.2). The direction of the electric current (*i* = 1.22 mA/cm^2^) was changed to the opposite of that used in the ED recovery of tartrates. The current density for cleaning step provided a sufficiently high salt flux from the former concentration compartment in the absence of water splitting.

Thus, DC (stage 1) became concentration compartment (stage 2) and vice versa CC_1_, CC_2_ (stage 1) became desalination compartments (stage 2).

The experiment lasted 1 h with periodic video recording, which was carried out through the end face of the laboratory ED cell. Every 5 min, the pH values and conductivity of the solution in the tank 12 were measured. After the end of the experiment, the solution from the former DC circuit was analyzed, and the surface of the CJMA-6 membrane was photographed. Then, the CVC of the CJMA-6 membrane was obtained using a fresh portion of model solution 4 in the DC circuit. The direction of the electric field was the same as in stage 1.

A similar cleaning experiment was carried out without applying an electric field.

### 2.4. Processing of ED Experimental Data

The degree of demineralization and concentration of the solution in the DC, CC_1_ and CC_2_ circuits was calculated by Formula (1):

(1) $$\gamma =\frac{\kappa_{t}-\kappa_{0}}{\kappa_{0}},\;$$

where $\kappa_{0}$ and $\kappa_{t}$ (μS/cm) are the initial value of the conductivity of the solution and its value after time t from the start of electrodialysis. A positive *″*γ*″* value indicates an increase in the concentration of ions in the CC circuits. Negative *″*γ*″* values indicate a removal of ions in the desalination circuit.

The degree of removal of tartrates (indicated by index T) from the desalination circuit was determined with Formula (2):

(2) $$\gamma_{T}=\frac{\left(c_{0}^{T}-c_{t}^{T}\right)}{c_{0}^{T}}$$

where $c_{0}^{T}$ and $c_{t}^{T}$ are the values of the total molar concentration of tartrates in the model solution before ED and after a time t from the start of electrodialysis.

The number of changes transported, in coulombs (C) was calculated with Formula (3):

(3) Q=I·t, 

where *I* is current (in A) and *t* is ED duration (in s).

The calculation of energy consumption (W·s or Joule) for the ED removal of tartrates was carried out according to Formula (4):

(4) W=I·Δφ·t, 

where Δφ is the potential drop between Luggin’s capillaries (in V) and *t* is ED duration (in s).

### 2.5. Estimation of Biofouling and Antioxidant Activity of Polyphenols

The antioxidant activity of solutions in the DC, CC_1_, and CC_2_ circuits during electrodialysis (stage 1) and membrane cleaning (stage 2) was estimated by the FRAP (Ferric Reducing/Antioxidant Power) [60] and ABTS [61] (2,2′-azino-bis-3-ethylbenzothiazoline-6-sulfonic acid) methods. The FRAP method [60] includes the reduction of Fe^3+^ ions to Fe^2+^ with antioxidants, the subsequent addition of a photometric reagent, and determination of the color intensity of this solution with the iron (II) complex. The concentration of antioxidants is directly proportional to the intensity of staining of the sample and is calculated using the calibration dependence. The essence of the ABTS method [61] is to obtain a colored solution with the ABTS^•+^ radical cation and reduce it to ABTS with compounds having antioxidant properties. Since the radical cation ABTS^•+^ is colored bluish-green, and ABTS is colorless, the concentration of antioxidants is determined by the difference in color intensity of these solutions. 

After steps 1 and 2, the samples were stored in containers with a solution of 0.005 M KCl at pH 3.25. The membranes were removed from the containers under sterile conditions. Scrapings from the surface of the membrane facing DC were carried out on a defatted glass slide. The obtained preparations were dried in air; fixed over an alcohol lamp flame; stained with fuchsine; and analyzed using an optical Primo Star microscope (Carl Zeiss, Oberkochen, Germany) with a set of 10×, 100× in the presence of immersion liquid.

## 3. Results and Discussion

### 3.1. ED Removal and Concentration of Components of Model Solutions

**Figure 3:**
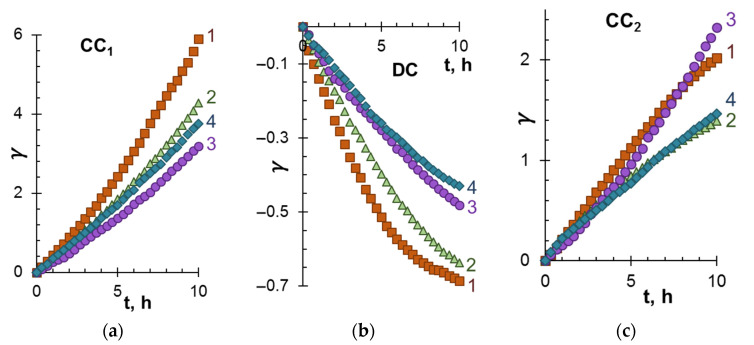
The degree of demineralization and concentration in the CC1 (**a**), DC (**b**) and CC2 (**c**) tracts vs. duration of electrodialysis of model solutions. The numbers of the curves correspond to the designations of the solutions in Table 2.

**Figure 4:**
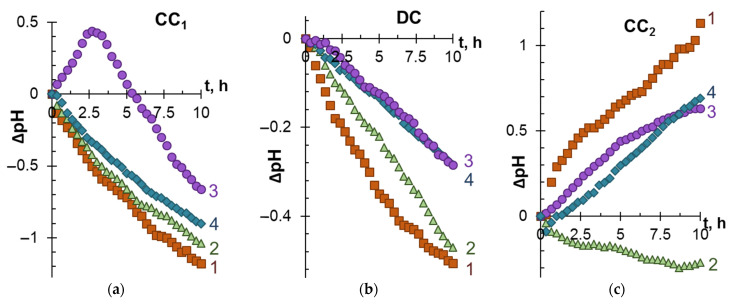
Differences between the current and initial pH values in the CC1 (**a**), DC (**b**) and CC2 (**c**) tracts vs. duration of electrodialysis of model solutions. The numbers of the curves correspond to the designations of the solutions in Table 2.

Figure 3 and Figure 4 present the dependencies of the degree of demineralization and concentration, as well as changes in the pH of model solutions in the DC and CC_1_, CC_2_ circuits during electrodialysis (stage 1).

As expected, with an increase in the ED duration, the conductivity of the solution in DC decreases, while in CC_1_ and CC_2_, on the contrary, it increases. The most significant demineralization is achieved in the case of model solution 1 (Figure 3b), which contains only KCl salts and species of tartaric acid. The addition of ethyl alcohol (solution 2) and especially polyphenols (solution 3) or polyphenols and fructose (solution 4) significantly impairs ED performances.

Accordingly, the highest values of *γ* for the CC_1_ circuit (Figure 3a) are observed in the case of solution 1. The value of *γ* noticeably decreases if the model solutions contain organic substances. A similar course of dependences *γ*–t characterizes the CC_2_ circuit. The difference with CC_1_ is observed in the case of model solution 3: in CC_1_, the *γ* values for this solution are minimal, while in CC_2_ they are comparable to *γ* for solution 1. It is important to note that in all cases considered, Ant and PACs remain in the desalination tract. Therefore, ED processing does not lead to any loss in the antioxidant activity of the model solutions 3 and 4.

When interpreting these data, it should be taken into account that cations K^+^ and H^+^ are transferred from DC to CC_1_. The mobility of H^+^, which contributes to the recorded conductivity of the solution, is an order of magnitude higher compared to the mobility of salt ions [57]. Therefore, the values of *γ* in CC_1_ turn out to be almost 2 times higher than in CC_2_. The transfer of protons to the CC_1_ tract causes acidification of the solution (Figure 4a). The complex nature of the dependence ΔpH–t (Figure 4a, curve 3) observed during the ED of model solution 3 is apparently caused by the participation of a part of protons in the protonation–deprotonation reactions of anthocyanins in desalination compartment [46]. A slight acidification of the model solutions in the DC tract during ED occurs due to the “acid dissociation” mechanism [62], which promotes the generation of protons by the CJMA-6 anion-exchange membrane at any electric current density (see Supplementary Materials, Figure S6).

The essence of the “acid dissociation” mechanism, which is described in detail in Supplementary Materials (Figure S7), is as follows. HT^−^ anions dissociate upon entering the anion-exchange membrane. Protons are excluded from CJMA-6 into the model solution due to Donnan exclusion. The formed doubly charged T^2−^ anions are transferred through the membrane into the CC_2_ solution. At the CJMA-6/solution interface faced to the CC_2_, T^2−^ anions interact with water to form HT^−^ and OH^−^ anions. The appearance of hydroxyl ions leads to alkalization of the solution in the CC_2_ circuit (Figure 4c). The presence of polyphenols in model solutions (which can also participate in protonation–deprotonation reactions [46,47]) or polyphenols and fructose in the model solutions reduces the generation of hydroxyl anions, but does not suppress it. It is interesting that in the case of model solution 2, not alkalization, but a slight acidification of the solution takes place in the CC_2_ tract. The nature of this phenomenon may be explained by the specific effect of ethyl alcohol on the mechanism of “acid dissociation”, the study of which is beyond the scope of our study.

**Figure 5:**
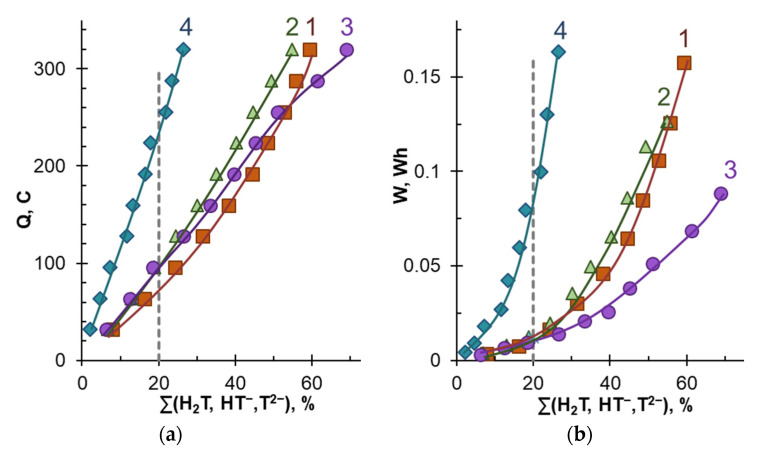
The number of electrical charges transported (**a**) and energy consumption (**b**) vs. the degree of tartrates removal from model solutions. The numbers near the curves correspond to the designations in Table 2. The dotted line corresponds to the degree of recovery, which is considered sufficient to stabilize red wines [47]. The solid lines should guide the eye.

Note, the phenomena caused by the protonation–deprotonation of tartaric acid species and polyphenols (namely, anthocyanins) generally do not prevent the transfer of tartaric acid anions to the CC_2_ tract. At the same time, the component composition of the model solution significantly affects the number of transported electric charges (*Q*) and required energy consumption (*W*) for tartrate stabilization of wine. Figure 5 shows the mentioned dependences. The dashed line indicates the degree of tartrates removal of 20%, which is considered sufficient to stabilize red wines [47]. Table 3 summarizes the number of electrical charges transported and energy consumption for ED removal of 20% tartrates from model solutions.

The shortest duration of electrodialysis and the smallest number of the electric charges transported are required for tartrate stabilization of model solutions that do not contain ethanol and potential foulants (polyphenols and fructose). The appearance of polyphenols in the model solution (solution 3) does not lead to a dramatic increase in *Q* values compared to those achieved in model solutions 1 and 2. Moreover, the energy consumption in the case of ED processing solution 3 decreases compared to solutions 1 and 2. This decrease is caused by the higher conductivity of solution 3 (Table 2) due to the presence of mineral salts (mainly KCl) in the extract from grape pulp, which was the source of Ant and PACs. The addition of fructose (model solution 4) causes a sharp deterioration in the energy parameters of electrodialysis. For example, for *γ_T_* equal to 20%, the *Q* and *W* values increase by factors of 2.4 and 7.1 compared to the number of electrical charges transported and the energy consumption in the case of solution 3.

The following sections will consider possible reasons for the observed deterioration in ED performance. 

### 3.2. The Mechanism of Membrane Fouling by Components of Model Solutions

**Figure 6:**
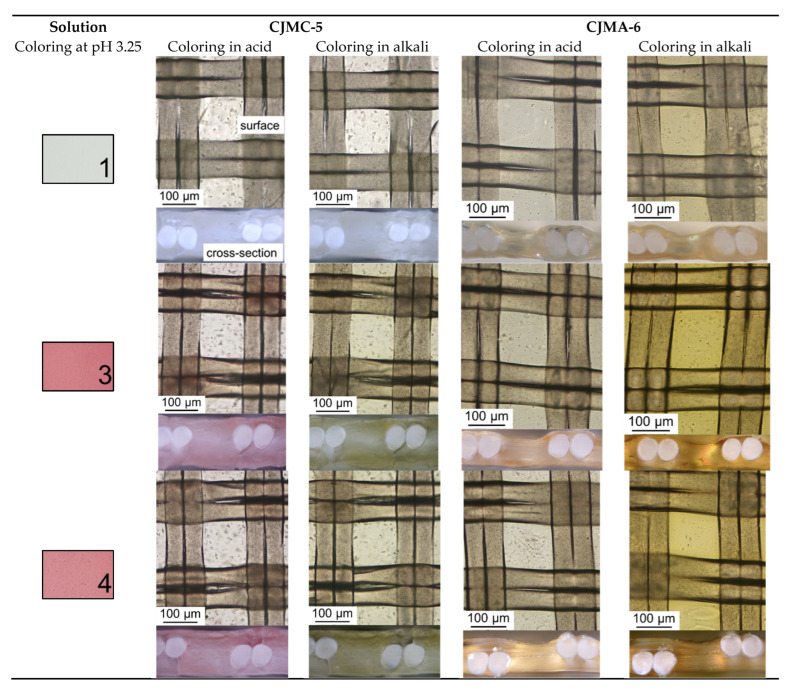

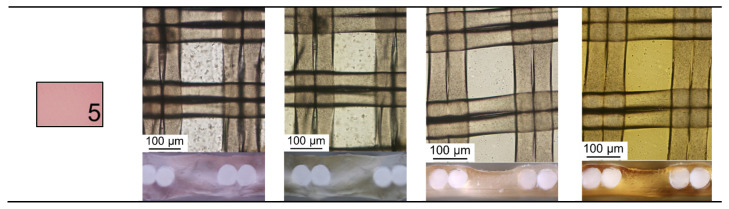
Optical images of the surface and cross-sections of CJMC-5 and CJMA-6 membranes after soaking in model solutions without applying an electric field (*i* = 0 mA/cm^2^). The numbers correspond to the designation of the model solutions in Table 2.

*Fouling in the absence of an electric field (i* = 0). The results of fouling of the studied membranes without applying an electric field (*i* = 0) should be discussed first before analyzing the fouling of CJMC-5 and CJMA-6 during ED. Figure 6 shows the surfaces and cross-sections of CJMC-5 and CJMA-6 after soaking for 10 h in model solutions 1, 3, 4 and 5 (Table 2). 

The color of the layer of these solutions with a thickness of 2 mm is shown in the same figure for comparison. The duration of soaking coincides with the duration of ED processing solutions 3 and 4. A drop of 0.1 M solutions of acid (NaOH) or alkali was applied to membrane samples before visualization to highlight the presence of anthocyanins. As already mentioned in the Introduction, these substances change color depending on the pH of the medium (see Figure S1). In an acidic medium, Ant are colored red. They are almost colorless in a neutral solution and become a yellow-brown in a highly alkaline medium. A detailed color scale of the model solutions depending on pH is shown in Supplementary Materials Figure S2. It was necessary to preliminarily enhance the color of anthocyanins after a relatively short time of soaking the CJMC-5 and CJMA-6 samples in the model solutions, since the concentration of anthocyanins in the membranes turned out to be low compared to that observed earlier in the case of aromatic materials [41,42,43]. Penetration of anthocyanins into the membranes took place through both surfaces in contact with the model solution.

As expected, the membrane samples practically did not change color after soaking in model solution 1 due to the absence of anthocyanins. The reason for the acquisition of a yellowish hue in a highly alkaline medium by sample CJMA-6, apparently, is the partial degradation of the anion-exchange material. Such degradation is characteristic of many anion-exchange membranes with ammonium fixed groups [63,64].

In the case of solution 5, which contains a minimum amount of minerals, staining of samples in an acidic medium is minimal. In an alkaline medium, a brown layer is visualized at the output of the threads of the reinforcing cloth to the surface. It points out that polyphenols most easily penetrate the membrane through the macropores between the ion-exchange material and the threads of the reinforcing cloth [43,55].

After soaking in solutions 3 and 4, cross-sections of membrane 3 in an acid medium become pinker. Moreover, the layers in the middle of the membrane volume are stained weaker. This means that in 10 h some of the low molecular weight Ant have time to occupy the entire volume of sample CJMC-5. At the same time, Ant in the composition of substances with a higher molecular weight are located near the surface. In an alkaline medium, these substances (complexes and/or colloidal particles) acquire a greenish color. Insignificant fouling by low-molecular anthocyanins is also observed for anion-exchange membrane CJMA-6. However, the layers near the surface of these samples contain significantly less high molecular weight polyphenols. The color shift of the cross-sections of the anion-exchange membrane to yellow compared to the pink-green color palette characteristic of the cation-exchange membrane is quite understandable.

These results are consistent with those obtained earlier [41,43] when studying the fouling of cation- and anion-exchange materials in cranberry juice [43], in wine [50] or in a model solution (pH = 3), which contained only polyphenols and mineral salts [41]. According to these studies, performed at *i* = 0, after a few hours, relatively small Ant species penetrate deep into cation-exchange materials, while larger PACs molecules are mainly adsorbed on their surface. Fouling is facilitated by electrostatic interactions between negatively charged fixed sulfonate groups of cation-exchange materials and anthocyanin cations (Ant+), which acquire a positive electrical charge at pH ≤ 3 [47]. Both PACs and Ant contain aromatic rings. Therefore, the adsorption of PACs on the surface of aliphatic CJMC-5 is largely caused by π-π (stacking) interactions with Ant that have penetrated the membrane. To a lesser extent, PACs interact with a small amount of aromatic rings of the crosslinking agent (sodium 4-styrenesulfonate), which is introduced at the final stage of CJMC-5 manufacturing [54]. Ant provide a bright red color of the volume of cation-exchange membranes and resins, and PACs on the surface give the color a burgundy hue.

On the contrary, at pH = 3 of the external solution, anion-exchange materials adsorb much less Ant compared to cation-exchange materials [39,41], because the pH of the internal solution in anion-exchange materials has pH ≈ 7 [41]. At this pH value, Ant are mainly in the purple quinoidal anhydrobase form [47]. This form has no electrical charge and does not enter into electrostatic interactions with positively charged fixed groups of anion-exchange resins or membranes. Indeed, a sufficiently high ion-exchange capacity of CJMA-6 (Table 1) contributes to a shift in pH to a neutral and even alkaline region. They remain almost colorless when acid is added before optical microscopy. The addition of alkali causes the transformation of all anthocyanins into yellow chalcon-anions. Thus, it can be assumed that Ant mainly penetrate into the CJMC-6 aliphatic anion-exchange membrane due to π-π (stacking) interactions with the aromatic rings of the crosslinker. Thus, the main reasons of the fouling phenomenon are π-π (stacking) interactions, formation of hydrogen bonds and electrostatic interactions. An aromatic material enhances π-π interactions and makes any cleaning less effective than in the case of an aliphatic material. High surface hydrophilicity provokes formation of hydrogen bonds between material and hydrophilic species of a solution. It should be also noted that high ion-exchange capacity of a material can lead to strong interactions with charged components of the solution. The contribution in the fouling process can be reduced by absence or negligible all or some of the listed phenomena. Thus, the use of aliphatic ion-exchange membranes with a low content of aromatic cross-linking agents allows us to hope for a decrease in their fouling by aromatic substances (anthocyanins and other polyphenols). The decrease in the ion-exchange capacity of membranes compared to traditionally used MK-40, CSE-fg [43] or AMX [50] reduces the hydrophilicity of membrane surface and electrostatic interactions between anthocianins and membranes. These circumstances allow us to hope for less fouling and easier cleaning in the case of using aliphatic membranes with a relatively low ion-exchange capacity for tartrate stabilization of wine.

**Figure 7:**
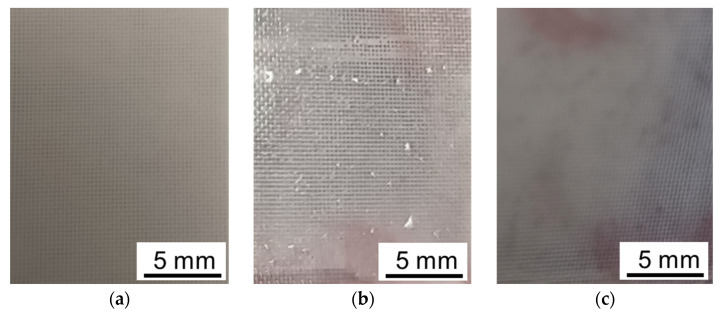
Photo of the surface of the CJMC-5 membrane before (**a**) and after its using in ED processing model solutions 3 (**b**) and 4 (**c**).

*Fouling in the electric field.* The results obtained after ED processing of model solutions make us hypothesize a special role of the applied electric field and tartrates in the fouling of CJMC-5 and CJMA-6 membranes. As expected, ED processing of solutions 1 and 2 has practically no effect on the appearance of the surfaces of the cation-exchange (CJMC-5) and anion-exchange (CJMA-6) membranes facing the desalination compartment. Surprisingly, only faint pink “islands” appeared on the CJMC-5 membrane samples after ED processing solutions 3 and 4 (Figure 7).

The color of these “islands” coincides with the color of dilute model solutions (Figure 6) and practically does not differ in the case of solution 3 (contains Ant and PACs) and in the case of solution 4 (contains Ant, PACs and fructose). Apparently, the staining indicates little adsorption of polyphenols (Ant and PACs) to those regions of the CJMC-5 cation-exchange membrane that contain a higher concentration of the aromatic cross-linker. Only microcavities on the surface of CJMC-5 (SM, Figure S8a), which could appear during membrane storage, have a more intense burgundy color. As in the absence of an electric field, these cavities, as well as outputs to the surface of CJMC-3 macropores between the ion-exchange material and the reinforcing cloth threads, serve as the main “gates” for the penetration of high-molecular-weight polyphenols into the membrane.

**Figure 8:**
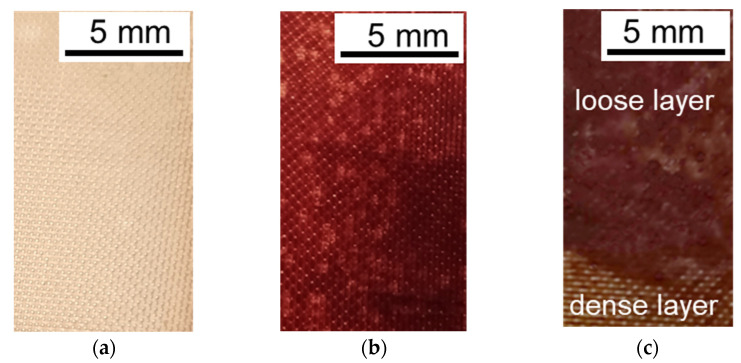
Surface photos and optical images of CJMA-6 membrane cross-sections before (**a**) and after ED processing of model solutions 3 (**b**) and 4 (**c**).

**Figure 9:**
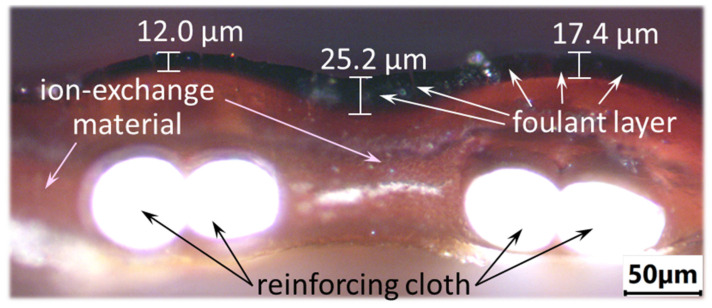
Optical image of a CJMA-6 membrane cross-section after ED processing of model solution 3 at a current density of 3.26 mA/cm^2^ during 36 h.

On the contrary, the entire surface of the CJMA-6 anion-exchange membrane is covered with a burgundy (Figure 8b) and brown (Figure 8c) sediment of colloidal particles, which contains Ant and PACs in the case of solution 3 or Ant, PACs and fructose in the case of solution 4. Moreover, some of the foulants penetrate deep into the membrane, coloring its volume fairly evenly (Figure S8b). Increasing the current density to 3.26 mA/cm^2^, as well as increasing the duration of ED up to 36 h, leads to the intense coloration of the entire volume of CJMA-6 and an increase in the thickness of the layer of maroon polyphenol sediment on its surface (Figure 9).

**Figure 10:**
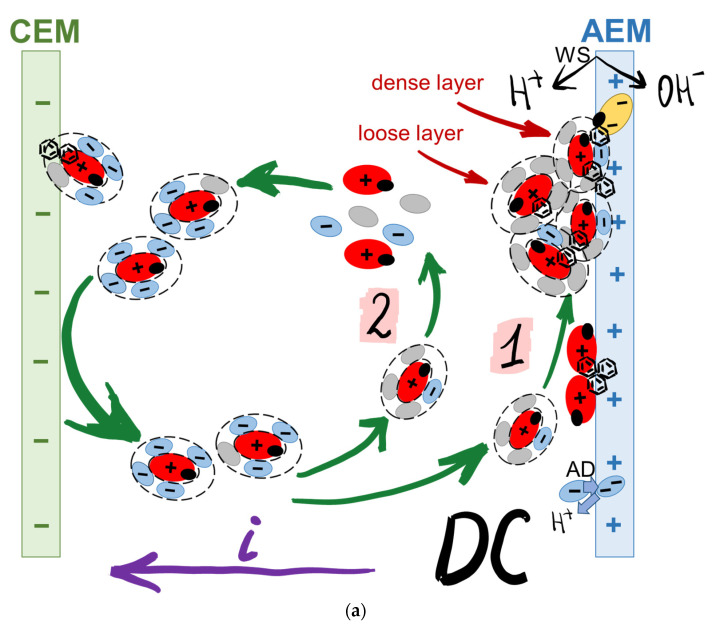

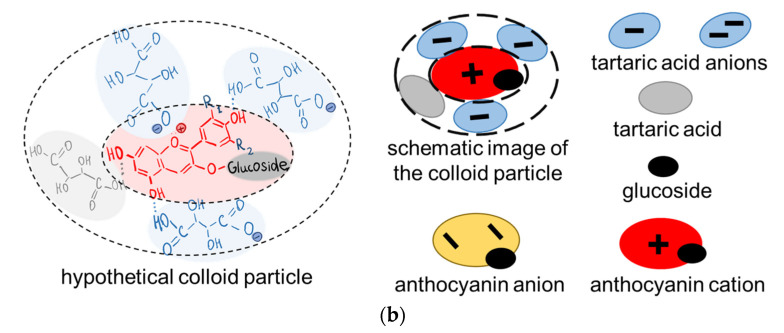
Hypothetical scheme of cation- and anion-exchange membranes fouling during ED of model solutions 3 and 4 (**a**), as well as the proposed structure and designation of colloid particles and other compounds (**b**). AD and WS are “acid dissociation” and water splitting mechanisms correspondingly; 1 shows a dense layer formation, 2 corresponds to a trap-mechanism formation of a loose layer.

Note that at pH = 3.25, which is maintained in all model solutions, anthocyanins should acquire a positive charge and be transferred by the electric field, not to the anion-exchange but to the cation-exchange membrane, which does not correspond to the observed data. Moreover, the “acid dissociation” phenomenon [62] causes additional acidification of the depleted solution near the surface of CJMA-6, which should enhance the electrostatic repulsion between Ant^+^ cations and CJMA-6 ammonium fixed groups, which have a positive charge. The following hypothesis (Figure 10) may explain the observed cation and anion-exchange membrane fouling during ED.

Tartaric acid species, the concentration of which is quite high in the studied model solutions, form hydrogen bonds with the hydroxyl groups of polyphenols (Figure 10a). In addition, Ant^+^ cations enter into electrostatic interactions with HT^−^ anions. The result of these interactions is the formation of colloid particles with a negatively charged surface (Figure 10b). These particles are repelled by the negatively charged surface of the cation-exchange membrane and, under the action of an electric field, are transferred to the surface of the anion-exchange membrane. 

Negatively charged colloidal particles enter into electrostatic interactions with positively charged fixed groups of CJMA-6 forming a dense layer of polyphenols on its surface. This dense brown layer (Figure 8c) is not mechanically removed from the surface of CJMA-6. Highly hydrated colloidal particles are attached to the dense layer by hydrogen bonding and π-π (stacking) interactions, forming a loose layer. The presence of fructose in the solution 4 promotes the formation of a loose layer due to the enhancement of hydrogen bonds formation.

The protons that are generated by the surface of CJMA-6 due to the “acid dissociation” mechanism react with some of the negatively charged colloid particles. Therefore, the particles become electrically neutral or are destroyed and acquire a positive electrical charge. These particles move towards the bulk of the solution due to the difference in concentration or due to electric field and meet with negatively charged HT^–^ anions. Thus, sediment accumulation on the surface of CJMA-6 follows the so-called “trap” mechanism. A similar phenomenon was observed, for example, when protein molecules move from the cation-exchange membrane to the bulk solution and back depending on the acquired electric charge under the action of an external electric field [65,66].

The sorption of anthocyanins by the volume of CJMA-6 follows the same mechanism as in the case of *i* = 0. In addition, it is possible that a certain amount of Ant^+^ in the composition of negatively charged colloidal particles penetrates into the pores of the CJMA-6 membrane [55], which are larger compared to the pores of CJMC-5 [49].

Water splitting, which enhances proton generation at the CJMA-6/diluted solution interface, appears to enhance the trap mechanism. As a result, the foulant layer on the surface of the anion-exchange membrane increases in the overlimiting current mode (Figure 9) compared to the underlimiting one (Figure 8).

The product of water splitting hydroxyl ions transferred through the CJMA-6 increases the pH of the internal solution. As a result, anthocyanins entering the membrane are converted into negatively charged yellow chalcon ions (Figure 10), which enhances the fouling of the anion-exchange membrane volume. Of course, this hypothesis needs to be refined and detailed, which we are going to do in further studies.

### 3.3. Current–Voltage Curves of the CJMA-6 Membrane before and after ED Processing of Model Solutions

**Figure 11:**
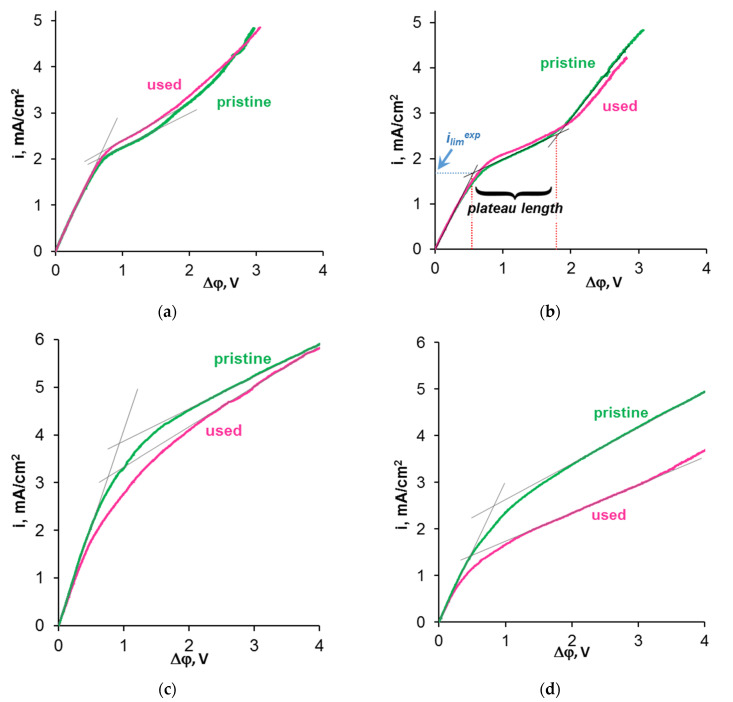
Current–voltage curves of the CJMA-6 membrane before (indicated as pristine) and after (indicated as used) ED processing of model solutions 1 (**a**), 2 (**b**), 3 (**c**) and 4 (**d**). The numbering of the solutions corresponds to Table 2.

Current–voltage curves of the CJMA-6 membrane (Figure 11) before and after ED processing of the model solutions give an idea of the effect of fouling on the membrane resistance and the phenomena caused by concentration polarization. The CVC characteristics, including the ohmic resistance of the CJMA-6 and adjacent solution layers, the “length” of the plateau, and the ratio of the limiting current values before and after ED processing of the model solutions, are summarized in Table 4. Standard procedure for determining the limiting current density (*i_lim_^exp^*) is shown at Figure 11b.

The CVC of a pristine membrane in model solution 1 (Figure 11a), which contains only a mixture of KCl and KHT, has the traditional shape described in much research [62,67,68,69]. The curve contains the initial section I, the section of the inclined plateau II and the “overlimiting” section III. The addition of ethyl alcohol (model solution 2) leads to an increase in the resistance of the initial section of the CVC (*R_p_ =* Δ*φ_I_*/*I*), a decrease in the value of *i_lim p_^exp^* by 18% and almost no effect on the “length” of the plateau (Figure 11b). These changes are to be expected. Indeed, *R_p_* is an integral value. The resistance of the membrane and the adjacent layers of the depleted and enriched solution, which are located between the Luggin’s capillaries, determine it. The decrease in *R_p_* (Table 4) is caused by a decrease in the conductivity of model solution 2 compared to solution 1 (Table 2). The decrease in *i_lim p_^exp^* in the case of model solution 2 is apparently caused by the suppression of electroconvection, which develops according to the “electroosmosis I” mechanism [70,71,72] due to a change in the dielectric constant of the depleted solution near the surface of CJMA-6.

The extract from grape pulp, which is added to model solution 2 to obtain solutions 3 and 4, contains some mineral salts (mainly KCl), in addition to polyphenols (Ant, PACs, etc.). An increase in the conductivity of the model solution 3 compared to the solution 2 and leads to a 1.87 times decrease in the *R_p_* value (Table 4). A growth of the experimental limiting current is caused by an increase in the anion concentration in model solutions 3 and 4 compared to solutions 1 and 2. In addition, the growth of *i_lim p_^exp^* in the case of solution 3 (Figure 11c) seems to be facilitated by the “island” adsorption of anthocyanins by the CJMA-6 surface (Figure 8b), which enhances the electroconvection. A similar phenomenon has been observed in the initial stages of anion-exchange membrane fouling with red wine [50]. On the contrary, shielding of the conductive surface of the anion exchange membrane, when added to fructose in model solution 4, leads to a decrease in *i_lim p_^exp^* and an increase in *R_p_* (Figure 11d). In both cases (Figure 11c,d), the “length” of the inclined plateau increases compared to solutions 1 and 2 because shielding of fixed groups on the surface of CJMA-6 by foulants inhibits water splitting.

The shape and parameters of the current–voltage curves remain practically unchanged after the operation of the CJMA-6 membrane in ED processing of model solutions 1 and 2. A small (about 10%) increase in *i_lim u_^exp^* compared to *i_lim p_^exp^* is most likely caused by partial destruction of the ion-exchange material, which causes the formation of cavities on the surface of CJMA-6. We observed a similar phenomenon [73] during ED desalination of a tartrate-containing solution using an AMX anion-exchange membrane (Astom, Tokyo, Japan). The caverns increase the geometric inhomogeneity of the CJMA-6 surface stimulating the development of electroconvection. In the case of ED processing of the model solution 3, *i_lim u_^exp^* decreases by 19% compared to *i_lim p_^exp^*, apparently due to screening of the conducting surface by the foulant. Shielding affects *i_lim p_^exp^* even more in the case of model solution 4 (Table 4) because the precipitate on the surface of CJMA-6 is denser and less permeable to anions that are transported through the membrane.

### 3.4. Cleaning of the Anion-Exchange Membrane in Operando

**Figure 12:**
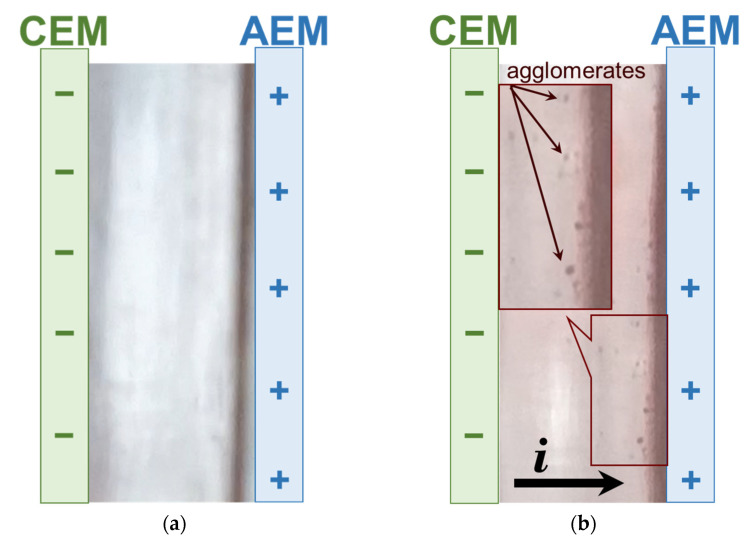
Frames from the video corresponding to the 10th minute of cleaning in operando without (**a**) and with (**b**) the use of an electric field (*i* = 1.22 mA/cm^2^). Cleaning was carried out after ED processing of model solution 4.

Figure 12 shows frames from the video (see SM) that correspond to the 10th minute of cleaning in operando (stage 2, Figure 1) in the absence of an electric field (*i* = 0) (SM, Video S1) and under an electric field (*i* = 1.22 mA/cm^2^) (SM, Video S2).

Pumping an acidified water–alcohol solution of KCl through the former (in stage 1) DC does not cause any visible changes if the electric field is absent (Figure 12a). On the contrary, the imposition of an electric field, the direction of which is opposite to that applied in step 1, leads to the occurrence of an unusual phenomenon. Quite large (several micrometers) foulant agglomerates detach from the surface of the CJMA-6 anion-exchange membrane. They slide along the CJMA-6/solution interface into the lower part of the compartment, are picked up by the pumped liquid flow, enter the bulk solution, and then are carried out of the intermembrane space into the intermediate tank 12. In the case of the CJMC-5 cation-exchange membrane, cleaning does not lead to any noticeable results.

**Figure 13:**
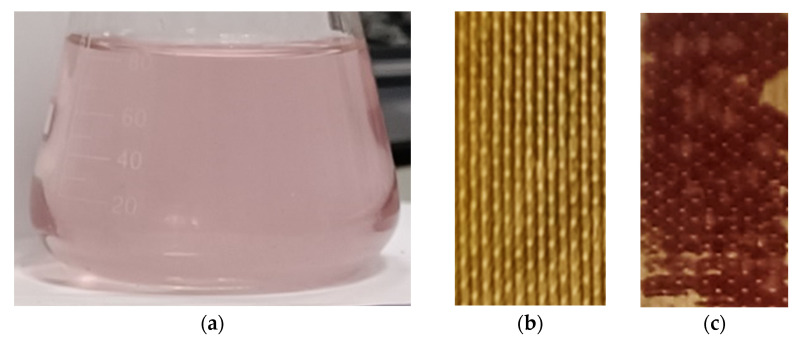
Photo of the cleaning solution (**a**) as well as the surfaces of the CJMA-6 anion-exchange membrane after cleaning in operando with (**b**) and without an electric field (**c**).

Note that cleaning in operando is accompanied by a 20% increase in the electrical conductivity as well as a slight (by 0.1 units) increase in pH of the solution in the former DC tract (which became the concentration tract). After stage 2, the cleaning solution turns pink (Figure 13a). Moreover, the ratio of polyphenols in the solution (14% Ant and 86% PACs) remains the same as in the initial model solution 4. The surface of CJMA-6 is largely cleared of sediment and becomes lighter (Figure 13b) compared to the photo obtained immediately after the electrodialysis removal of tartrates from model solution 4 (Figure 8c). The limiting current on the CVC of the cleaned membrane increases by 9% compared to that measured after electrodialysis. At the same time, the sample that was not exposed to electric current did not undergo any significant changes (Figure 13c).

Apparently, in the case of CJMA-6, a change in the direction of the electric current during cleaning led to the transport of Cl^–^ anions from the adjacent compartment. A significant increase in Cl^–^ concentration at the foulant layer/solution interface caused the destruction of hydrogen bonds between the components of colloidal particles due to the well-known effect of “salting out” [74]. The gravitational force compelled the formed conglomerates of colloidal particles to move down, and the forced convection of the solution finally “tore off” them from the surface of CJMA-6. The change in the polarity of the electric field at the stage of cleaning contributed to this “detachment” and migration transfer of negatively charged colloidal particles from the membrane surface into the bulk of the solution. Therefore, the membrane was fouled by the model solutions’ components; however, this fouling was reversible due to the short processing time and the aliphatic membrane matrix. Thus, such membranes are more promising for ED processing of polyphenol containing solutions compared to membranes with aromatic matrix, which are currently more popular.

**Figure 14:**
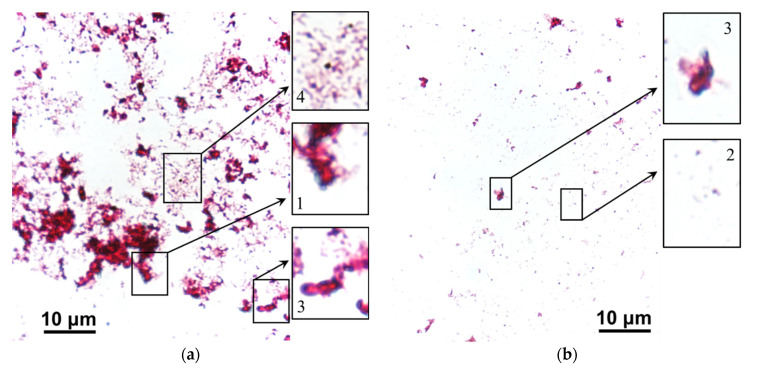
Stained scrapings from the surface of CJMC-5 cation-exchange membrane after cleaning in operando without (**a**) and with (**b**) electric field application. Legend: 1 is bacterial conglomerates, 2 is bacterial cells, 3 is fungal conglomerates, 4 is rod-shaped bacteria.

**Figure 15:**
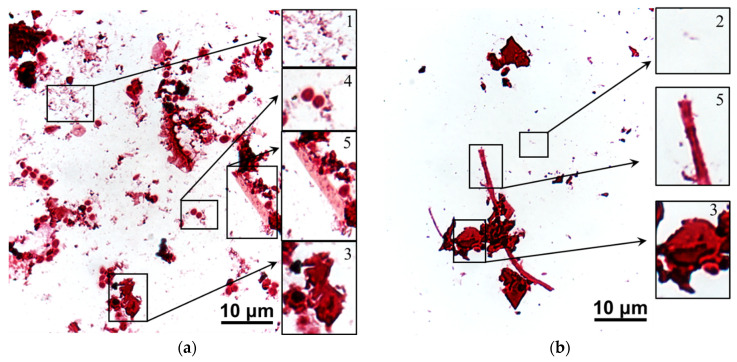
Stained scrapings from the surface of CJMA-6 anion-exchange membrane after cleaning in operando without (**a**) and with (**b**) electric field application. Legend: 1 is bacterial conglomerates, 2 is bacterial cell, 3 is fungal conglomerate, 4 is fungal spores, 5 is fungal mycelium.

Figure 14 and Figure 15 show images of stained scrapings from the surfaces of ion-exchange membranes that were used in ED processing of model solution 3 and then were cleaned in operando with and without an electric field (blank experiment).

After a blank experiment, large accumulations of dead bacterial and fungal conglomerates were found on the fouling surfaces of both (CJMC-5, CJMA-6) ion-exchange membranes. The rod-shaped forms of bacteria were most abundant on the CJMC-5 membrane (Figure 14), while fungal spores and destroyed mycelium were found on the CJMA-6 membrane (Figure 15).

After cleaning in operando using an electric field, only single bacterial cells were observed on both membranes (Figure 14 and Figure 15); moreover, actinomycetes predominated in the case of the anion-exchange membrane. Regardless of the cleaning method, more active biofouling occurs in the case of an anion-exchange membrane. As many authors have repeatedly reported [75,76], AEM biofouling is facilitated by the presence of fixed amino groups, which are nutrients for microorganisms. Anthocyanins, proanthocyanins and fructose, which are not removed from the surfaces of CJMC-5 and CJMA-6 membranes, can also serve as a nutrient medium in the case of cleaning without applying an electric field. On the contrary, cleaning in operando using an electric field deprives microorganisms of their nutrient base, preventing their reproduction. The cleaning method tested is probably the first attempt to use a constant continuous electric field in combination with environmentally friendly reagents to extend the life of aliphatic ion-exchange membranes used in the production of wine and juice.

## 4. Conclusions

The successive complication of the composition of the model solutions used for the electrodialysis removal of tartrates made it possible to analyze the effect of ethyl alcohol, polyphenols (mainly anthocyanins and proanthocyanidins) and saccharides (fructose) on the fouling of aliphatic ion-exchange membranes CJMA-6 and CJMC-5 (manufacturer Hefei Chemjoy Polymer Materials Co. Ltd., Hefei, China).

The main contribution to the increase in energy consumption is made by the appearance of fructose in the model solution. Moreover, the mechanisms of fouling of the studied aliphatic membranes are different under the conditions of the flow of electric current and in its absence. In particular, under the conditions of electrodialysis carried out in the underlimiting current mode, the fouling of the CJMC-5 cation-exchange membrane with polyphenols and fructose is negligible. On the contrary, anthocyanins penetrate into the CJMA-6 anion exchange membrane, and colloidal particles are deposited on its surface. The thickness of the layer of colloidal particles grows in the overlimiting current mode and with an increase in the duration of electrodialysis.

The hypothesis explaining the unusual behavior of aliphatic membranes suggests that π-π (stacking) interactions of polyphenols with the ion-exchange matrix do not play a decisive role in this case. Fouling is more strongly influenced by electrostatic interactions and the formation of hydrogen bonds between anthocyanins, proanthocyanidins, fructose and tartaric acid particles, which cause the formation of colloidal particles with a negatively charged surface. These particles are repelled from the negatively charged surface of the CJMC-5 cation exchange membrane, but enter into electrostatic interactions with positively charged fixed groups on the surface of the CJMA-6 anion exchange membrane. The electric field promotes migration delivery of negatively charged colloid particles to CJMA-6.

The deposition of a loose layer of colloid particles on top of a dense layer is carried out due to the implementation of a known for protein fouling trap mechanism. In our case, tartaric acid species and protons are involved in the trap mechanism. Protons appear at the CJMA-6/diluted solution interface due to the deprotonation of tartaric acid particles after their entry into the membrane and the Donnan exclusion of protons into the external solution (“acid dissociation” mechanism). In overlimiting current modes, water splitting promotes proton entry into the depleted solution, causing increased fouling.

A successful attempt was made to clean CJMA-6 in operando by pumping an aqueous-alcoholic solution of KCl through the desalination compartment and changing the polarity of the constant continuous electric field compared to the electrodialysis stage. This cleaning promotes the destruction of colloid particles on the surface of the aliphatic anion exchange membrane. In addition, it inhibits the biofouling of ion-exchange membranes by depriving microorganisms of the food supply.

In the future, this approach can also be used for the selective extraction of polyphenols with high antioxidant activity. After refinement (determining the optimal current modes, the duration of the electric field application, improving the composition of the cleaning solution), cleaning in operando can compete with chemical methods using acids, alkalis, oxidizing agents or mixtures of water with organic solvents, which are dangerous for the environment.

## Figures and Tables

**Table 1:** Some characteristics of the membranes used in the experiments [43,50,55].

Designation	Fixed Group	Matrix Material	Membrane Thickness in 0.02 M NaCl Solution, µm	Water Uptake, g_H2O_/g_dry_, %	Ion-Exchange Capacity, mmol/g_wet_
CJMA-6	–N+ (CH_3_)_3_	Polyolefin	120 ± 3	18 ± 1	0.90 ± 0.05
CJMC-5	–SO_3_^2–^	Polyvinylidene fluoride	154 ± 3	32 ± 5	0.57 ± 0.07
MA-41	–N+ (CH_3_)_3_, –N+ (CH_3_)_2_, –N+ (CH_3_)	Cross-linked polystyrene with divenylbenzene	450 ± 50	33 ± 3	1.22 ± 0.06

**Table 2:** Designations and composition of model solutions.

Solution Designation	KCl, M	^1^ K_x_H_(2−x)_T, M	C_2_H_5_OH, %	Polyphenols, mg/L		Fructose, g/L	pH	κ, mS/cm
				Ant	PACs			
1	0.005	0.013	-	-	-	-	3.25	2.040
2			10	-	-	-		1.493
3				20	130	-		2.790
4						1.0		2.080
5	-	-	10	20	130	-		0.935

^1^—a mixture of potassium dihydrotartrate (KHT), potassium tartrate (K_2_T) and tartaric acid (H_2_T) in the ratio of mole fractions 0.63:0.06:0.31.

**Table 3:** Some characteristics of ED processing of the studied model solutions.

Solution Designation	The Degree of Tartrates Removal, %	Electrodialysis Duration, s	The Number of Changes Transported, C	Energy Consumption 103, W h
1	20 ± 2	9000	79 ± 5	13 ± 1
2		11,580	94 ± 5	13 ± 1
3		11,520	99 ± 5	12 ± 1
4		27,120	235 ± 5	85 ± 1

**Table 4:** Some parameters of the current–voltage curves of the CJMA-6 anion-exchange membrane obtained in model solutions before (index p) and after (index u) electrodialysis.

Solution Designation	* *R_p_*, Ω cm^2^	*R_u_/R_p_*	*i_lim p_ ^exp^*, mA/cm^2^	*i_lim u_^exp^/i_lim p_^exp^*	“Length” of the Plateau _p_, V	“Length” of the Plateau _u_, V
1	2.78	1.00	2.01	1.08 ± 0.2	1.28	1.15
2	2.80	1.06	1.68	1.11 ± 0.2	1.25	1.44
3	2.20	1.08	4.00	0.81 ± 0.2	>4.22	>4.22
4	2.84	1.03	2.37	0.59 ± 0.2	>5.11	2.93

* Ohmic resistance of CJMA-6 and adjacent solution layers at I → 0.

## Data Availability

Not applicable.

## Supplementary Materials

The following supporting information can be downloaded at: https://www.mdpi.com/article/10.3390/membranes12121187/s1, Figure S1: The structures of anthocyanins and their modifications depending on the pH of the medium; Figure S2: Effect of pH on the color of model solutions 5 (a) and 3 (b); Figure S3: Schematic structure of individual anthocyanins and proanthocyanidins (PACs); Figure S4: The structure of fructose; Figure S5: The structure of tartaric acid; Figure S6: Speciation diagrams: distribution of the tartaric acid species (in mole fractions) vs. the pH of the solution; Figure S7: Scheme of generation of H+ and OH– ions caused by “acid dissociation” mechanisms in the systems AEM/tartrate containing solution; Figure S8: Optical images of CJMC-5 (a) and CJMA-6 (b) membrane surfaces (indicated by number 1) and cross-sections (indicated by number 2) after ED processing of solution 3. Images were taken in transmitted light; Table S1: The detailed composition of the model solutions and red wine; Video S1: The process of cleaning in operando without applying current density (I = 0); Video S2: The process of cleaning in operando with applying current density (I = 1.22 mA/cm^2^). References [77,78,79,80,81,82] are cited in the supplementary materials.
