# Supplementary material for: Electrodialysis Tartrate Stabilization of Wine Materials: Fouling and a New Approach to the Cleaning of Aliphatic Anion-Exchange Membranes
Source: Membranes (Basel). 2022 Nov 25;12(12):1187. doi: 10.3390/membranes12121187 (PMC9785266; doi:10.3390/membranes12121187)
Supplement: Supplementary file 1 [file membranes-12-01187-s001.zip › 4 Supplementary materials Fouling and cleaning.pdf]

Supplementary materials

Electrodialysis tartrate stabilization of wine materials: fouling and a new approach to the cleaning of aliphatic anion-exchange membranes

Tsygurina K., Pasechnaya E., Chuprynina D., Melkonyan K., Rusinova T., *Nikonenko V., Pismenskaya N.

1. Structures of some components of wines and the model solutions

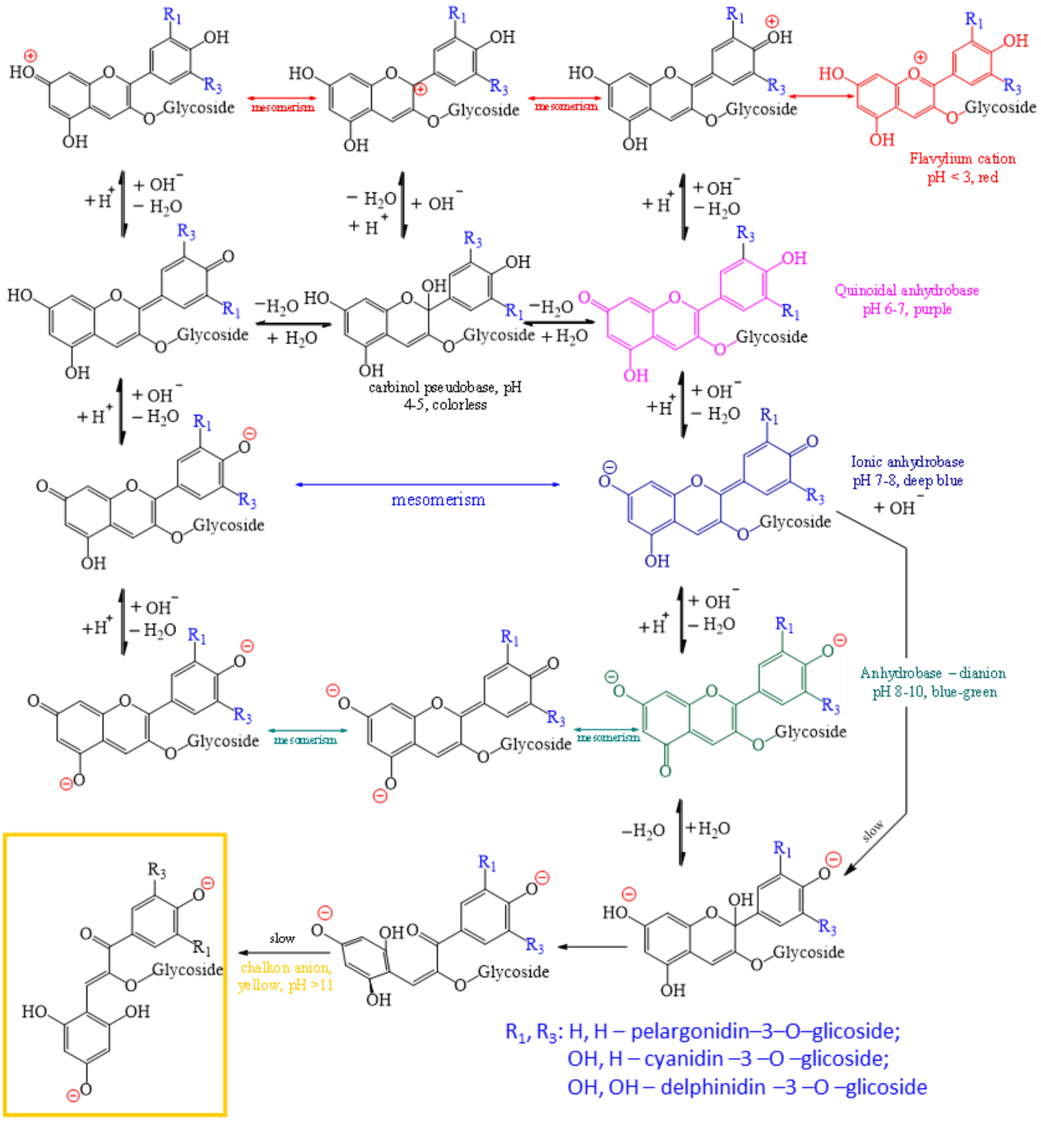

Figure S1. The structures of anthocyanins and their modifications depending on the pH of the medium. Adapted from [1]. *R1*, *R3* are —H, or —OH, or —OCH3 groups; *Glycoside* is glucose, rhamnose, arabinose or galactose.

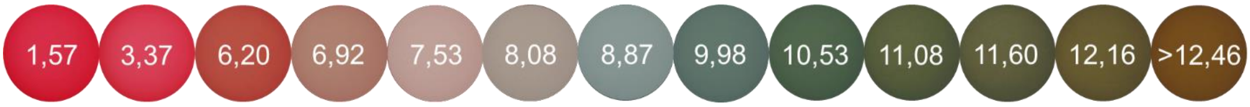

a

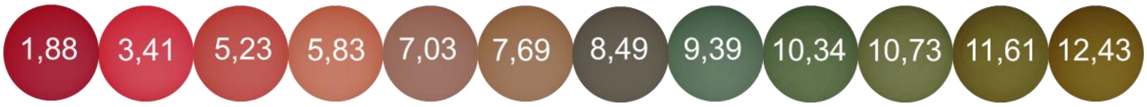

b

Figure S2. Effect of pH on the color of model solutions 5 (a) and 3 (b). Designation of the solutions corresponds to Table 2 in main text. The pH values of the solutions are indicated for each color.

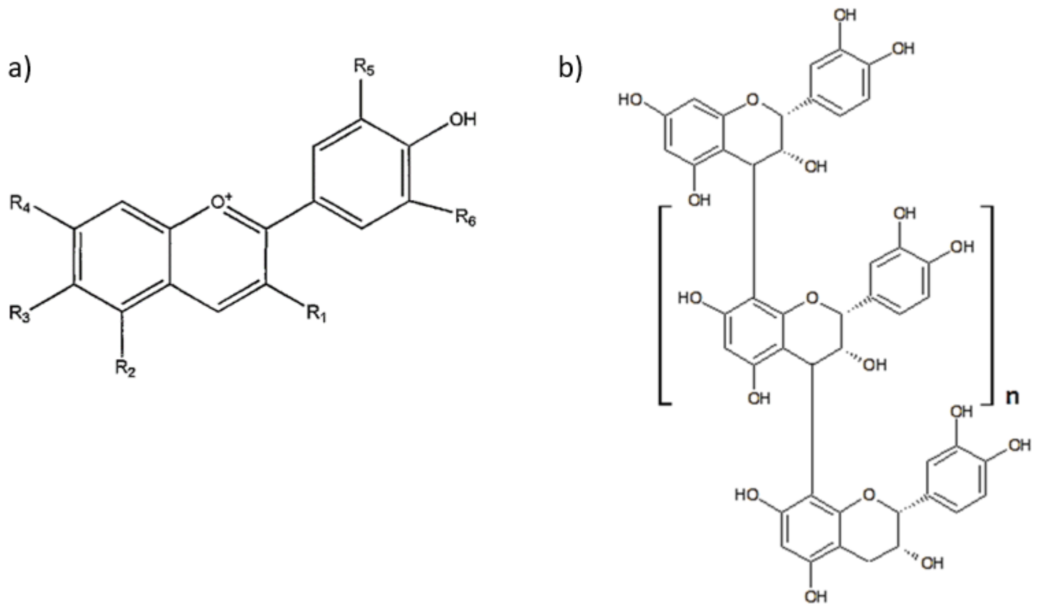

Figure S3. Schematic structure of individual anthocyanins and proanthocyanidins (PACs) [2,3].

Fructose is a ketonic simple sugar with the formula $C_6H_{12}O_6$. According to IUPAC nomenclature fructose is (–)-D-arabino-hex-2-ulopyranose. In the solid state and in solutions fructose exists in cyclic hemiacetal forms at room temperature with next distribution[4].

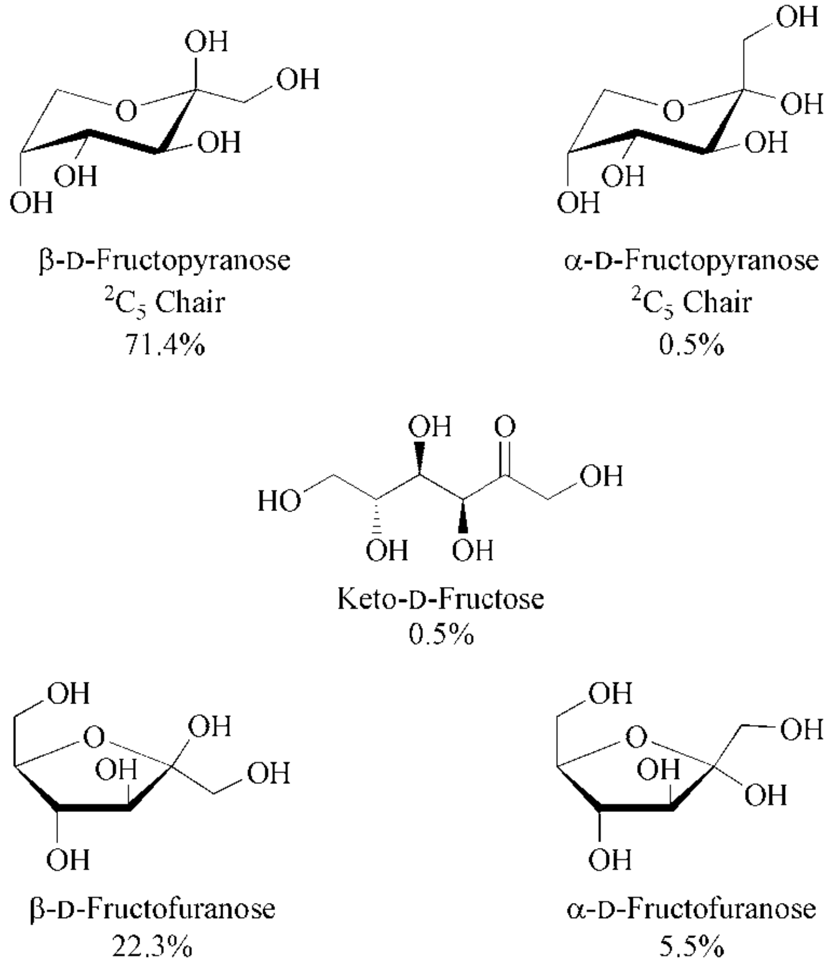

Figure S4. The structure of fructose.

Hydrogen tartrate ion, $C_4H_5O_6^-$, denoted by HT^-, is a singly charged anion of the tartaric acid (H_2T). The IUPAC name of H_2T is 2,3 - dihydroxybutanedioic acid, $C_4H_6O_6$, which has the structure presented in Figure S5.

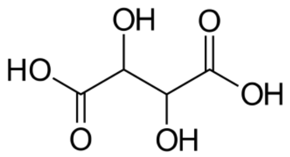

Figure S5. The structure of tartaric acid.

Figure S6 shows the distribution of species of the polybasic acids under study (in mole fractions) vs. the pH of the solution. These distributions are calculated using the appropriate equilibrium equations and the pK_a values presented in the figure.

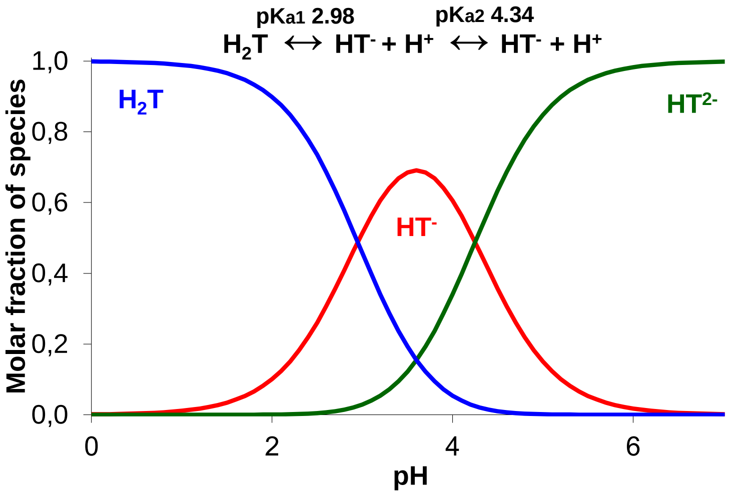

Figure S6. Speciation diagrams: distribution of the tartaric acid species (in mole fractions) vs. the pH of the solution [5].

2. The detailed composition of the model solutions and average composition for dry red wines

The detailed composition of the model solutions and one red wine are presented in Table S1.

Table S1. The detailed composition of the model solutions and red wine (for comparison).

**Component**	***Model solutions**				****Wine**
	**No 1**	**No 2**	**No 3**	**No 4**	
	**g/L**	**g/L**	**g/L**	**g/L**	**g/L**
**ANIONS**					
Chloride	0.20	0.20	0.22	0.22	not determined
Sulphate	-	-	0.48	0.48	not determined
**ORGANIC ACIDS**					
Tartrate	2.00	2.00	2.00	2.00	not determined
Malate	-	-	0.24	0.24	not determined
Succinate	-	-	0.14	0.14	not determined
Citric	-	-	0.15	0.15	not determined
Lactate	-	-	0.21	0.21	not determined
Total	2.00	2.00	2.74	2.77	3.50-15.0
**CATIONS**					
Potassium	0.20	0.20	0.86	0.86	0,5-1,5
Sodium	-	-	0.08	0.08	0.03-0.05
Magnesium	-	-	0.10	0.10	0.05-0.15

Calcium	-	-	0.08	0.08	0.05-0.15
**POLYPHENOLS**					
Anthocyanins	-	-	0.20	0.020***	≤0.50****
Proanthocyanidins, Tanins, etc.	-	-	0.13	0.13	0.19-3.80
Carbohydrates:<br>total<br>fructose	-	-	-	not determined<br>1.00	1.50-3.80<br>1.00-2.00
**ALCOHOLS**					
Ethyl alcohol	-	78.9	78.9	78.9	72.0-120.0

*Data obtained using chromatographic methods, capillary electrophoresis; anthocyanins and proanthocyanidins were determined spectrophotometry methods;
**Data from Ribéreau-Gayon et al. [1] for dry red wines
***eq. cyanidin 3-glucoside
****eq. gallic acid

3. *"Acid dissociation" mechanism*

The charge of the anion, which is the acid residue, increases in absolute value after the detachment of H^+ ion, which makes it possible to transfer a larger number of charges through the membrane by the same number of anions. The mechanism is described below and presented at Figure 7.

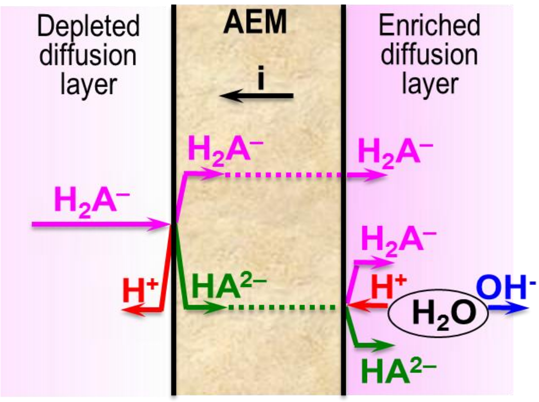

Figure S7. Scheme of generation of H^+ and OH^- ions caused by "acid dissociation" mechanisms in the systems AEM/tartrate containing solution [2].

The reason for the dissociation of singly-charged anions is the fact that H^+ ions are co-ions for the AEM. They are excluded from the membrane due to the Donnan effect [6], and this leads to the fact that the pH of the internal solution in the AEM is 1.5-3 pH units higher than the pH of the external solution[7]. Higher pH values of the internal solution cause deprotonation of the weak acid anions when they enter the membrane. As a result, part of the charge is transferred in the AEM by doubly-charged (or even triply-charged) anions. When leaving the membrane into an enriched solution, doubly-charged anions again enter a more acidic environment, which determines their partial protonation (Fig. S6). The result of this process is the generation of OH^- ions at the membrane/enriched solution boundary. Thus, the processes of generation of H^+ and OH^- ions in ampholyte-containing membrane systems are separated in space[8]: the generation of H^+ ions occurs at the depleted solution/AEM interface, and OH^- ions are generated at the AEM/enriched solution interface.

4. Results of membrane fouling study by optical spectroscopy

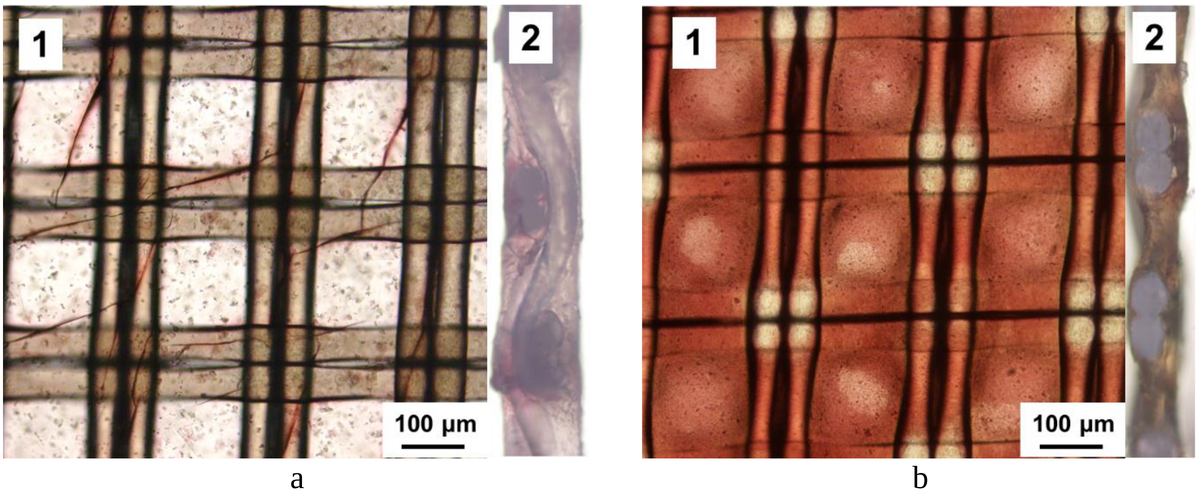

Figure S8. Optical images of CJMC-5 (a) and CJMA-6 (b) membrane surfaces (indicated by number 1) and cross-sections (indicated by number 2) after ED processing of solution 3. Images were taken in transmitted light.

The process of cleaning *in operando* with and without applying current density was recorded by using a digital camera and presented at files named as Video S1 (i=0) and Video S2 (i>0).

References

1. Ribéreau-Gayon, P.; Glories, Y.; Maujean, A.; Dubourdieu, D. *Handbook of Enology, Volume 2: The Chemistry of Wine - Stabilization and Treatments,*; 2nd-nd Editi ed.; 2006; ISBN 978-0-470-01038-9.
2. Czochanska, Z.; Foo, L.Y.; Newman, R.H.; Porter, L.J. Polymeric Proanthocyanidins. Stereochemistry, Structural Units, and Molecular Weight. *J. Chem. Soc. Perkin Trans. 1* **1980**, 2278–2286, doi:10.1039/P19800002278.
3. Khoo, H.E.; Azlan, A.; Tang, S.; Lim, S. Anthocyanidins and Anthocyanins: Colored Pigments as Food, Pharmaceutical Ingredients, and the Potential Health Benefits. *Food Nutr. Res.* **2017**, *61*, 1361779, doi:10.1080/16546628.2017.1361779.
4. Wach, W. Fructose. In; 2004 ISBN 9783527306732.
5. Rybalkina, O.A.; Sharafan, M.V.; Nikonenko, V.V.; Pismenskaya, N.D. Two Mechanisms of H+/OH− Ion Generation in Anion-Exchange Membrane Systems with Polybasic Acid Salt Solutions. *J. Memb. Sci.* **2022**, *651*, 120449, doi:10.1016/j.memsci.2022.120449.
6. Helfferich, F. *Ion Exchange*; McGraw-Hill: New York: NY, USA, 1962; ISBN 0-486-68784-8.
7. Kozmai, A.E.; Nikonenko, V.V.; Zyryanova, S.; Pismenskaya, N.D.; Dammak, L. A Simple Model for the Response of an Anion-Exchange Membrane to Variation in Concentration and PH of Bathing Solution. *J. Memb. Sci.* **2018**, *567*, 127–138, doi:10.1016/j.memsci.2018.07.007.
8. Rybalkina, O.; Tsygurina, K.; Melnikova, E.; Mareev, S.; Moroz, I.; Nikonenko, V.; Pismenskaya, N. Partial Fluxes of Phosphoric Acid Anions through Anion-Exchange Membranes in the Course of NaH2PO4 Solution Electrodialysis. *Int. J. Mol. Sci.* **2019**, *20*, doi:10.3390/ijms20143593.
